# Review: biological engineering for nature-based climate solutions

**DOI:** 10.1186/s13036-022-00287-8

**Published:** 2022-03-29

**Authors:** Benjamin R. K. Runkle

**Affiliations:** Department of Biological and Agricultural Engineering, University of Arkansas, Fayetteville, USA

**Keywords:** Soil carbon, Climate change, Engineering curriculum, Emissions avoidance, Diversity and inclusion

## Abstract

Nature-based Climate Solutions are landscape stewardship techniques to reduce greenhouse gas emissions and increase soil or biomass carbon sequestration. These mitigation approaches to climate change present an opportunity to supplement energy sector decarbonization and provide co-benefits in terms of ecosystem services and landscape productivity. The biological engineering profession must be involved in the research and implementation of these solutions—developing new tools to aid in decision-making, methods to optimize across different objectives, and new messaging frameworks to assist in prioritizing among different options. Furthermore, the biological engineering curriculum should be redesigned to reflect the needs of carbon-based landscape management. While doing so, the biological engineering community has an opportunity to embed justice, equity, diversity, and inclusion within both the classroom and the profession. Together these transformations will enhance our capacity to use sustainable landscape management as an active tool to mitigate the risks of climate change.

## Background

Global climate change impacts all human systems and landscapes [1], causing long-term changes of ecological function [2] and an economic cost more than twice current global gross domestic product [3]. While the primary way to prevent and slow these changes is to decarbonize the energy supply by reducing fossil fuel energy sources [4, 5], landscape-management solutions have a role to play and often deliver win-win successes on multiple metrics, beyond just climate [6]. In the United States their potential is equivalent to one-fifth of current net annual emissions [7]. These Nature-based Climate Solutions (NbCS) either harness the photosynthetic power of ecosystems to store carbon in soils and vegetation, or they reduce existing emissions of greenhouse gases from agricultural and other managed landscapes. Soil carbon storage strategies include peatland restoration, forest agriculture, residue retention and cover cropping, and increased photosynthesis [8]. Emissions reductions strategies in landscapes often focus on non-CO_2_ greenhouse gases such as N_2_O (via nutrient management [9]) and CH_4_ (through modified rice irrigation [10–12] or changes in cattle management [13]). Whole-farm and life-cycle or supply-chain approaches extend these concerns to reduce fuel use or otherwise limit CO_2_ emissions, though the variety of implementation strategies and paucity of data means that realistic depictions of NbCS in life cycle assessments remain challenging [14].

NbCS strategies provoke new ways of thinking about land management [15], policy-making [16], economic bookkeeping [17], and leadership [18], but in doing so introduce potential issues that must be addressed. While there is a large body of evidence that NbCS approaches work [19, 20], there is also considerable uncertainty in the range of achievable sequestration or emissions reduction, in appropriate management tools, and in generalizing findings from one set of conditions to another [21, 22]. Thus, questions remain on how to measure and predict land-atmosphere carbon exchange, soil carbon storage, and reduced emissions. Many NbCS will bring environmental co-benefits like biodiversity, sustainable food production, and improved water quality [23]. However, others may bring harms such as increased labor, fire risk, and unequitable societal impacts (e.g., environmental racism). There is also much to be learned about resource management with, and from, indigenous communities [24]; this insight includes fire management, traditional agriculture, and ways of knowledge.

Resolving the “wicked challenge” of multi-objective landscape management that includes climate and other co-benefits [25] will require new types of education for a new workforce, and training for existing workers. The biological engineering profession can lead this transition [26], as it is both uses applied science (i.e., engineering) and is an applied discipline (taking engineering concepts to living systems), blending elements of ecological engineering [27] with soil or landscape management and agricultural engineering [28, 29]. Work in a systems perspective at the interface of these disciplines is needed for challenges from climate change to agriculture [30, 31] and the resultant obligation to create a productive, resilient, and proactive “climate-smart agriculture” system [32]. The critical engagement of researchers in education regarding new metrics of what determines successful landscape management (e.g., regenerative agriculture, or sustainable intensification) is essential to both guide effective solutions and clearly communicate them to the public [33, 34]. Thus, in this essay, I bring attention to NbCS in a biological engineering context, highlighting research needs, new application areas, methods of education, and attention to diversity, inclusion, and equity.

## Research, design, and education needs and new directions

### Overview and directions

To develop and implement sustainable NbCS strategies, new engineering research is needed to build lasting and nimble socio-technical change [35]. Research needs include (1) designing new measurement tools (e.g., fully using artificial intelligence techniques, novel sensor development, etc. [36, 37]), (2) providing application expertise on how to control and optimize landscape performance, and (3) translating that knowledge into action by landscape managers who need help prioritizing conservation activities among other landscape benefits (such as food provision). This research must be reflective and iterative, willing to account for new innovations, drivers of societal or geographical change, and user or implementer needs [38]. Finally, these social and research needs should invite pedagogical reflection: how can these ideas be embedded into the classroom experience, and how can all students and learners be included?

### Design new measurement tools and systems

Skills associated with biological engineering are needed to optimize measurement platforms at all sizes and domains to monitor landscape and management decisions and to guide individual decision making. At the large scale, to quantify and certify carbon capture or emission reductions methods, innovative multi-scale measurement platforms are needed [39]. Among many satellite observations and tools [40] we now have daily, 1-m satellite imagery (e.g., from Planet Labs) that provides an extraordinary, and under-utilized, opportunity to track land cover changes, land use dynamics, and plant response to weather or management [41, 42]. Application areas include mapping crop yield [43, 44], stress detection [45], and guiding precision agriculture [46]. However, these measurement and monitoring systems must be validated with technically challenging, on-the-ground measurements that require tailored instrumentation for each site. Popular research-scale approaches to better constrain carbon cycle processes in real time include eddy covariance [47, 48] and solar-induced chlorophyll fluorescence [49, 50]. Better planning and advance modeling can increase the information gained by the deployment of these systems [51], and so can networked science [52–55].

Expertise is needed to reduce the time or technical commitment to measurements and to inform farm or landscape-level interventions. Slow, point-based estimates of leaf area index, a critical parameter for understanding landscape conditions, can be translated into estimates over larger spatial extents from RGB images on UAVs [56] or satellite products [57], or from models based on days after planting or degree day [58]. Work is still needed for challenging but essential measurements such as soil moisture or rice field inundation that are difficult to remotely sense due to canopy coverage and poor penetration within the soil [59]. However, both measures are critical for understanding the permanence of soil carbon [60] and the rate of CH_4_ emissions from rice fields [12]. At the farm scale, engineering skills are needed for creative approaches to low-cost tools to support field-level interventions. For example, new and inexpensive redox-sensitive films may guide water management in rice production or wetland or marsh restoration [61, 62]. User-facing decision support tools can help weigh a suite of options across a set of target metrics [63–65] and provide verification of measurable environmental impacts [66].

### How to optimize the landscape across multiple services

What applications of biological engineering are there to resolve the challenge of implementing sustainable NbCS’s? A suite of new technologies is needed to sequester CO_2_ as soil organic carbon to enable both food and climate security – these include advanced rock weathering, agronomic interventions, and developing high yielding crops [67] – and they all must be tuned to their landscape and socio-economic setting. An agronomic intervention with quickly growing research interest and an open-ended solution is in the design and maintenance of a site- and application-appropriate microbiome. Whether through microbial or nutrient amendment, or other care, a healthy microbiome has potential to boost soil carbon conservation and raise crop yields [68, 69]. New experiments and tools, as well as fundamental knowledge on microbial communities and plant-microbe interactions, are needed to capture the diversity of possible benefits from expanded understanding of the microbiome [70–72].

Microbiome development and maintenance can support the larger material economy through the reuse of animal waste or plant residue. The American Society of Agricultural and Biological Engineers (ASABE) has engaged members with the circular economy as a means to transform food and agriculture systems, including to “regenerate natural systems” [73, 74]. While circular systems advocacy still needs to put a greater emphasis on biodiversity, this approach can boost soil carbon sequestration among a large range of ecosystem services [75, 76]. Circular economy methods on the landscape typically imply application of animal or plant waste products back onto the field for enhanced organic matter and field performance. The methods thus help reduce reliance on energy-intensive extraction for field applications of inorganic fertilizer while enhancing soil carbon inputs. How to do this well, and in different socio-economic contexts, is still challenging, and engineering design research and systems thinking are needed to find creative circular bioeconomy and bioproduct solutions [77, 78].

### Translate ideas into action into action

Biological engineering expertise is needed in many areas of translating research and design approaches into practical NbCS strategies. Expertise is needed to advise and assess projects for effective climate finance of agricultural emissions reductions [79] or the use of green microfinance [80]. Land-based climate mitigation strategies are already cost-effective in many cases (up to 40%), and additional potential exists if costs can be lowered through technical innovation [81]. Biological and agricultural engineers can work to increase agricultural input use efficiency to complement other sustainability initiatives and regenerative practices in the food and agriculture sector [82]. Land use management and conservation is necessarily iterative [83], and the engineering disciplines work well in this setting – improving, testing, and innovating towards optimal solutions.

Implementation of NbCS depends on many enabling factors from states, communities, and actors, and in perceived reliability [84]. In my group’s research assessing the sustainability of different rice management techniques, we have also seen the benefit of social networks, as farmers can compare notes and encourage each other toward more efficient implementation [64]. This finding is consistent with other studies that demonstrate the value of training, networking, and motivation on practice implementation [85, 86]. Practitioners want to know—is this problem really the biggest priority? Often, life cycle assessments can help deliver that message in a more holistic manner, grounding solutions within a quantifiable framework. Fortunately, new developments in life cycle assessments can improve representations of soil carbon storage [87, 88], forest carbon cycling [89], and sustainable rice production practices [90]. Moreover, engineers can work with designers to develop and improve management tools to better convey best options and alternatives, with clear depictions of uncertainty, risk, cost, and benefit along a variety of decision metrics.

### Curricular and program changes

To train the workforce and advance research towards sustainable NbCS implementation, new forms of education are needed alongside curriculum revision. In addition to embedding NbCS throughout the biological engineering curriculum, we must also provide tools for lifelong learners through cooperative extension, community outreach, and other channels. Within a biological engineering program, there are many ways to engage with NbCS, particularly as these programs are often taught in land-grant or similar universities with a tradition of place-based research, access to field research stations and expertise. As biological engineering degree programs have a variety of names and subject areas [91] and integrate many competencies [92] focusing on living systems [93], there is no one-size fits all solution for bringing NbCS into the classroom. However, in all these cases, coursework materials can be developed around the landscape (or other living systems) and their role in climate change solutions. While my home department is “Biological and Agricultural Engineering”, I encourage readers affiliated with Biomedical Engineering programs to also consider these topics in the context of their curriculum. Those issues could include the mental and physical health benefits of being near nature [94], or the potential impact of climate change on biomedical and public health interventions and outcomes [95]. Similarly, bioprocess engineering programs could continue work on higher-value products from traditional food and agricultural waste streams [96].

Engaging students with classroom learning strategies that mimic real-world problem-solving is one recommendation from a recent review of biological engineering education [97]. With this need in mind, I am working to develop a cross-campus undergraduate honors course whose central project would be an outline for Nature-based Climate Solutions strategy for my state, Arkansas, assessing scientific potential and political-economic opportunities. This project-oriented focus recognizes that localized and place-based environmental examples can enhance educational engagement and are also practical, taking advantage of local student knowledge and creating potentially impactful societal outcomes [98, 99]. In the course we would assess opportunities in Arkansas’s varied landscapes, including row crops, forestry, pasture, and urban areas. In each landscape type there will be scientific uncertainties, political and economic realities, and social expectations to contend with. In most geographic places—i.e., around any educational setting or university—there are a variety of landscapes in close proximity where students may have direct familiarity. An aim for the class is to motivate real change via letters on a state plan to the governor’s office or for the campus to increase its carbon neutrality efforts [100].

Curricular changes could enable NbCS education by revising both mandatory course materials and modifying elective course options. Recognizing the many skills needed to generate and sustain NbCS approaches, emphasis can be expanded on areas such as ecosystem ecology, plant ecophysiology, human decision making, agricultural and resource economics, modeling, or remote sensing that propel understanding of our landscapes and their spatio-temporal dynamics. The importance of NbCS can be integrated into existing Biological Engineering topic areas, such as green stormwater infrastructure, which can be designed to better sequester carbon in addition to its primary, stormwater retention and filtration roles [101, 102]. Methods courses which teach appropriate tools (such as Geographic Information Systems; GIS) or the code of ethics and the role of the engineer in society could use NbCS as the motivating example. Similarly, carbon accounting or the uncertainties associated with landscape change could be included within engineering economics instruction. My home department, among others, has emphasized sustainable food, water, and energy (following their well-studied ‘nexus’ [103, 104]); elevating carbon or climate to this list would help demonstrate its equal importance in course and degree design.

Landscape carbon management and NbCS could then be integrated into design and topic area courses, encouraged for design projects from first to final year, and curated to involve engagement with real clients and stakeholders while also meeting professional expectations. Professors of cooperative extension could come into the classroom as guest lecturers, demonstrating case studies of successful interventions and behavior change among landscape managers and agricultural producers [105]. In turn students may be attracted to this profession, which is known to provide economic and agricultural benefits to rural and other areas [106]. In the classroom, a complex systems perspective could be explored [107], emphasizing the challenge of balancing among different ecosystem services, trade-offs, and their economics, and teaching different types of problem-solving and presentation. In many cases these topics would provide instruction towards the Fundamentals of Engineering exam and eventual licensure. Capstone courses designed around the contemporary and complex challenges associated with NbCS could also be used to meet ABET accreditation requirements and desired student outcomes [108].

Beyond curricular change and shifts in higher education strategies, there is more work that engineering societies can do to enhance research and implementation of NbCS. NbCS needs are referenced only within “enhancing photosynthesis for agricultural productivity” in a recent discussion of emerging issues in biological engineering [109], and deep discussions of the role of biological engineering to play against climate change were missing. However, there are strong foundations on which to build: ASABE has a technical community on “Natural Resources & Environmental Systems”, and the Journal of Biological Engineering has “Ecological and environmental engineering” as a topic area. Together these outlets can push for further engagement on NbCS progress.

### Justice, diversity, equity, and inclusion

Designing innovative NbCS strategies requires the creative involvement, knowledge, and lived experience of all people [110]. All peoples and cultures have experienced nature—and the management of landscapes—in different ways. We need to incorporate perspectives on the landscape—and on perceived solutions—from indigenous and other under-represented groups. There are landscape management and sustainability lessons to be learned from women [111], indigenous peoples [112, 113], and historically marginalized racial groups [114]. These communities must be incorporated into implementation plans, ensuring fair access to incentive programs and feedback on the co-benefits and negative effects of land management changes [115]. Diverse perspectives on land stewardship can also inform the classroom environment, by encouraging different perspectives and methods of learning, by bringing in diverse voices and readings, and by supporting indigenous communities on campus [116]. Training that includes more representation in the classroom—via inclusive curriculum design or other ways to hear diverse voices—can also create a more inclusive environment [117, 118], helping provide long-term benefits by diversifying the workforce. There have been many calls to diversify professions traditionally associated with landscape management that lack diverse representation [119], and intentionally remove obstacles that prevent full participation [120]. Implementing tested retention strategies such as mentorship, inclusion in high impact academic experiences, and re-training faculty on intentional inclusion can also help create a diverse and representative future workforce [121].

## Conclusions and future perspectives

Mitigating climate change will alter our energy systems and landscapes and will alter the trajectory of our built and managed environments alike. Using our managed landscapes to respond to climate change is an opportunity to pursue alongside changes in the energy sector, but we must act quickly to develop research agendas, workforce training, and implementation strategies, as NbCS are slower and less direct than energy sector decarbonization approaches [122]. We will need the development of new tools, new approaches, and new education strategies. Like NbCS themselves, many of these initiatives would have spin-off co-benefits, advancing fundamental knowledge on landscape conservation practices, integration of other disciplines (from artificial intelligence to geography or political economy) into biological engineering, and active learning in the classrooms.

## Data Availability

Not applicable.

## Abbreviations

ASABE: American Society of Agricultural and Biological Engineers
CH_4_: methane
CO_2_: carbon dioxide
NbCS: Nature-based Climate Solutions
N_2_O: nitrous oxide
RGB: red green blue
